# 1-Methyl-4-(1-methyl-1*H*-benzimidazol-2-yl)pyridinium iodide

**DOI:** 10.1107/S1600536809053938

**Published:** 2009-12-19

**Authors:** Fang-Ming Wang

**Affiliations:** a School of Materials Science and Engineering, Jiangsu University of Science and Technology, Zhenjiang 212003, People’s Republic of China

## Abstract

The cation of the title compound, C_14_H_14_N_3_^+^·I^−^, is non-planar, the dihedral angle between the benzimidazole and the 1-methyl­pyridinium planes being 37.4 (2)°. The crystal structure is stabilized by weak π–π stacking inter­actions, the centroid–centroid distances between 1-methyl­imidazole and benzimidazole planes being 3.678 (4) Å.

## Related literature

For background to imidazole and its derivatives, see: Huang *et al.* (2004 ▶). For the biological activity of benzimidazole, see: Demirayak *et al.* (2002 ▶); Pawar *et al.* (2004 ▶). 
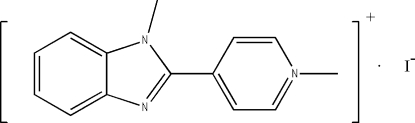


## Experimental

### 

#### Crystal data

C_14_H_14_N_3_^+^·I^−^
*M* *_r_* = 351.18
Triclinic, 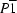

*a* = 7.7048 (15) Å
*b* = 9.9264 (18) Å
*c* = 10.1772 (19) Å
α = 64.888 (3)°
β = 72.933 (3)°
γ = 76.394 (4)°
*V* = 668.2 (2) Å^3^
*Z* = 2
Mo *K*α radiation
μ = 2.38 mm^−1^
*T* = 291 K
0.35 × 0.25 × 0.05 mm

#### Data collection

Bruker SMART CCD area-detector diffractometer
Absorption correction: multi-scan (*SADABS*; Bruker, 2000 ▶) *T*_min_ = 0.493, *T*_max_ = 0.887
3353 measured reflections
2307 independent reflections
1840 reflections with *I* > 2σ(*I*)
*R*_int_ = 0.058

#### Refinement

*R*[*F* ^2^ > 2σ(*F* ^2^)] = 0.043
*wR*(*F* ^2^) = 0.092
*S* = 1.00
2307 reflections
163 parameters
H-atom parameters constrained
Δρ_max_ = 0.94 e Å^−3^
Δρ_min_ = −0.48 e Å^−3^

### 

Data collection: *SMART* (Bruker, 2000 ▶); cell refinement: *SAINT* (Bruker, 2000 ▶); data reduction: *SAINT*; program(s) used to solve structure: *SHELXTL* (Sheldrick, 2008 ▶); program(s) used to refine structure: *SHELXTL*; molecular graphics: *SHELXTL*; software used to prepare material for publication: *SHELXTL*.

## Supplementary Material

Crystal structure: contains datablocks I, global. DOI: 10.1107/S1600536809053938/bx2252sup1.cif

Structure factors: contains datablocks I. DOI: 10.1107/S1600536809053938/bx2252Isup2.hkl

Additional supplementary materials:  crystallographic information; 3D view; checkCIF report

## Figures and Tables

**Fig. 1.**
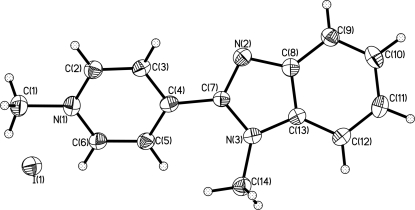
The asymmetric unit of the title compound with atom labels. Displacement ellipsoids were drawn at the 30% probability level.

## supplementary crystallographic information

### Comment

Imidazole and its derivatives are a very important kind of heterocyclic compounds with N-donor atoms, therefore they can be excellent organic ligands to generate various complexes (Huang *et al.*, 2004). Owing to its biological activities such as antimicrobial, antifungal (Pawar *et al.*, 2004), anticancer (Demirayak *et al.*, 2002), and so on, benzimidazoles have also received much attention. The construction of new member of this family is an important direction in the development of modern coordination chemistry and biological chemistry. We report here the synthesis and crystal structure of the title compound. The molecular structure is shown in Fig. 1. The cation of (I) is non-planar, the dihedral angle between the benzimidazolyl plane and the *N*-methylpyridinium plane is 37.4 (2)°. The crystal structure is stabilized by π–π [Cg1: N2-C7-N3-C13-C8; Cg2(i): C8/C13, code symmetry: (i) -x+2, -y+1, -z] stacking interaction, the distance centroid-centroid between these planes is  3.678 (4) Å. The crystal packing also exhibits a weak intermolecular C—H···I interaction.

### Experimental

Metallic sodium (0.25 g, 10.8 mmol) was dissolved in the stirred anhydrous ethanol(10 ml) under an atmosphere of nitrogen. Then added 2-(4-pyridinyl)-1*H*-benzimidazole (1.95 g, 10 mmol), dry actone (150 ml) and methyl iodide(1.24 ml, 20 mmol) in the above solution. The reaction mixture was refluxed for 24 h. When the reaction stopped and the mixture were cooled to room temperature, the solution were removed under decompression. Then the residue recrystallized from water twice to obtain the single crystals (3.0 g, 8.66 mmol).

### Refinement

All H atoms were fixed geometrically and were treated as riding on their parent C atoms, with C—H distances in the range of 0.93—0.96 Å, and with *U*_iso_(H) = 1.2*U*_eq_(parent atom), or *U*_iso_(H) = 1.5*U*_eq_(C_methyl_).

### Crystal data

**Table:**

C_14_H_14_N_3_^+^·I^−^	*Z* = 2
*M**_r_* = 351.18	*F*(000) = 344
Triclinic, *P*1	*D*_x_ = 1.745 Mg m^−^^3^
Hall symbol: -P 1	Mo *K*α radiation, λ = 0.71073 Å
*a* = 7.7048 (15) Å	Cell parameters from 1236 reflections
*b* = 9.9264 (18) Å	θ = 3.1–22.1°
*c* = 10.1772 (19) Å	µ = 2.38 mm^−^^1^
α = 64.888 (3)°	*T* = 291 K
β = 72.933 (3)°	Piece, colorless
γ = 76.394 (4)°	0.35 × 0.25 × 0.05 mm
*V* = 668.2 (2) Å^3^	

### Data collection

**Table:**

Bruker SMART CCD area-detector diffractometer	2307 independent reflections
Radiation source: sealed tube	1840 reflections with *I* > 2σ(*I*)
graphite	*R*_int_ = 0.058
phi and ω scans	θ_max_ = 25.0°, θ_min_ = 2.3°
Absorption correction: multi-scan (*SADABS*; Bruker, 2000)	*h* = −9→7
*T*_min_ = 0.493, *T*_max_ = 0.887	*k* = −10→11
3353 measured reflections	*l* = −12→12

### Refinement

**Table:**

Refinement on *F*^2^	Primary atom site location: structure-invariant direct methods
Least-squares matrix: full	Secondary atom site location: difference Fourier map
*R*[*F*^2^ > 2σ(*F*^2^)] = 0.043	Hydrogen site location: inferred from neighbouring sites
*wR*(*F*^2^) = 0.092	H-atom parameters constrained
*S* = 1.00	*w* = 1/[σ^2^(*F*_o_^2^) + (0.0301*P*)^2^] where *P* = (*F*_o_^2^ + 2*F*_c_^2^)/3
2307 reflections	(Δ/σ)_max_ < 0.001
163 parameters	Δρ_max_ = 0.94 e Å^−^^3^
0 restraints	Δρ_min_ = −0.48 e Å^−^^3^

### Special details

**Table:**

Geometry. Least-squares planes (*x*,*y*,*z* in crystal coordinates) and deviations from them (* indicates atom used to define plane)7.5483 (0.0040) *x* + 0.9256 (0.0225) *y* + 1.1206 (0.0247) *z* = 6.5511 (0.0171)* 0.0017 (0.0039) N1 * 0.0018 (0.0041) C2 * -0.0063 (0.0040) C3 * 0.0074 (0.0040) C4 * -0.0041 (0.0042) C5 * -0.0004 (0.0041) C6Rms deviation of fitted atoms = 0.00446.8822 (0.0088) *x* + 3.4760 (0.0239) *y* + 7.0324 (0.0184) *z* = 8.6547 (0.0229)Angle to previous plane (with approximate e.s.d.) = 37.43 (0.15)* -0.0020 (0.0031) C7 * -0.0067 (0.0031) N2 * 0.0128 (0.0031) C8 * -0.0138 (0.0030) C13 * 0.0098 (0.0030) N3Rms deviation of fitted atoms = 0.0100
Refinement. Refinement of *F*^2^ against ALL reflections. The weighted *R*-factor *wR* and goodness of fit *S* are based on *F*^2^, conventional *R*-factors *R* are based on *F*, with *F* set to zero for negative *F*^2^. The threshold expression of *F*^2^ > σ(*F*^2^) is used only for calculating *R*-factors(gt) *etc*. and is not relevant to the choice of reflections for refinement. *R*-factors based on *F*^2^ are statistically about twice as large as those based on *F*, and *R*- factors based on ALL data will be even larger.

### Fractional atomic coordinates and isotropic or equivalent isotropic displacement parameters (Å^2^)

**Table:**

	*x*	*y*	*z*	*U*_iso_*/*U*_eq_	
C1	0.7976 (9)	0.1720 (7)	0.3724 (7)	0.0574 (18)	
H1A	0.7850	0.1418	0.4774	0.086*	
H1B	0.9149	0.1293	0.3311	0.086*	
H1C	0.7027	0.1374	0.3557	0.086*	
C2	0.7960 (8)	0.4044 (7)	0.1519 (7)	0.0443 (15)	
H2A	0.8121	0.3452	0.0977	0.053*	
C3	0.7870 (8)	0.5551 (6)	0.0807 (7)	0.0422 (15)	
H3A	0.7948	0.5992	−0.0214	0.051*	
C4	0.7660 (8)	0.6442 (6)	0.1610 (6)	0.0387 (14)	
C5	0.7505 (8)	0.5736 (6)	0.3135 (6)	0.0422 (15)	
H5A	0.7337	0.6297	0.3707	0.051*	
C6	0.7598 (8)	0.4226 (7)	0.3784 (7)	0.0445 (15)	
H6A	0.7506	0.3759	0.4806	0.053*	
C7	0.7700 (8)	0.8070 (6)	0.0779 (6)	0.0339 (13)	
C8	0.8426 (8)	1.0150 (6)	−0.0938 (6)	0.0395 (14)	
C9	0.9152 (8)	1.1306 (7)	−0.2232 (7)	0.0443 (15)	
H9A	1.0063	1.1104	−0.2983	0.053*	
C10	0.8473 (9)	1.2748 (7)	−0.2354 (7)	0.0498 (17)	
H10A	0.8941	1.3536	−0.3202	0.060*	
C11	0.7082 (9)	1.3076 (7)	−0.1230 (7)	0.0482 (16)	
H11A	0.6644	1.4071	−0.1361	0.058*	
C12	0.6369 (8)	1.1955 (6)	0.0047 (7)	0.0423 (15)	
H12A	0.5454	1.2161	0.0793	0.051*	
C13	0.7076 (7)	1.0494 (6)	0.0175 (6)	0.0344 (13)	
C14	0.5260 (8)	0.8957 (7)	0.2645 (7)	0.0478 (16)	
H14A	0.5225	0.7909	0.3245	0.072*	
H14B	0.4090	0.9405	0.2400	0.072*	
H14C	0.5541	0.9438	0.3187	0.072*	
I1	0.26309 (6)	0.26836 (5)	0.37099 (4)	0.05037 (19)	
N1	0.7821 (6)	0.3387 (5)	0.2996 (6)	0.0432 (12)	
N2	0.8779 (7)	0.8606 (5)	−0.0548 (5)	0.0426 (12)	
N3	0.6668 (6)	0.9141 (5)	0.1277 (5)	0.0367 (11)	

### Atomic displacement parameters (Å^2^)

**Table:**

	*U*^11^	*U*^22^	*U*^33^	*U*^12^	*U*^13^	*U*^23^
C1	0.062 (5)	0.042 (4)	0.064 (5)	−0.010 (3)	−0.008 (4)	−0.019 (3)
C2	0.047 (4)	0.049 (4)	0.045 (4)	−0.011 (3)	−0.011 (3)	−0.023 (3)
C3	0.043 (4)	0.046 (4)	0.042 (4)	0.001 (3)	−0.011 (3)	−0.022 (3)
C4	0.030 (3)	0.047 (4)	0.042 (4)	−0.001 (3)	−0.008 (3)	−0.021 (3)
C5	0.046 (4)	0.045 (4)	0.042 (4)	−0.010 (3)	−0.007 (3)	−0.022 (3)
C6	0.053 (4)	0.044 (4)	0.037 (3)	−0.004 (3)	−0.008 (3)	−0.018 (3)
C7	0.032 (3)	0.039 (3)	0.033 (3)	−0.005 (3)	−0.008 (3)	−0.016 (3)
C8	0.043 (4)	0.042 (4)	0.039 (3)	−0.006 (3)	−0.016 (3)	−0.016 (3)
C9	0.045 (4)	0.046 (4)	0.043 (4)	−0.011 (3)	−0.010 (3)	−0.016 (3)
C10	0.052 (4)	0.051 (4)	0.046 (4)	−0.020 (3)	−0.021 (3)	−0.005 (3)
C11	0.048 (4)	0.038 (4)	0.065 (5)	−0.001 (3)	−0.024 (4)	−0.021 (3)
C12	0.041 (4)	0.044 (4)	0.048 (4)	0.001 (3)	−0.013 (3)	−0.024 (3)
C13	0.032 (3)	0.041 (4)	0.036 (3)	−0.003 (3)	−0.014 (3)	−0.017 (3)
C14	0.042 (4)	0.047 (4)	0.053 (4)	−0.004 (3)	−0.008 (3)	−0.021 (3)
I1	0.0493 (3)	0.0535 (3)	0.0478 (3)	−0.0021 (2)	−0.0128 (2)	−0.0197 (2)
N1	0.034 (3)	0.039 (3)	0.057 (3)	−0.003 (2)	−0.013 (3)	−0.017 (3)
N2	0.045 (3)	0.046 (3)	0.044 (3)	−0.006 (2)	−0.014 (3)	−0.021 (2)
N3	0.031 (3)	0.045 (3)	0.038 (3)	−0.004 (2)	−0.009 (2)	−0.020 (3)

### Geometric parameters (Å, °)

**Table:**

C1—N1	1.490 (8)	C8—N2	1.389 (7)
C1—H1A	0.9600	C8—C13	1.397 (8)
C1—H1B	0.9600	C8—C9	1.397 (8)
C1—H1C	0.9600	C9—C10	1.368 (8)
C2—N1	1.343 (7)	C9—H9A	0.9300
C2—C3	1.353 (8)	C10—C11	1.412 (9)
C2—H2A	0.9300	C10—H10A	0.9300
C3—C4	1.395 (7)	C11—C12	1.368 (8)
C3—H3A	0.9300	C11—H11A	0.9300
C4—C5	1.386 (7)	C12—C13	1.388 (7)
C4—C7	1.475 (8)	C12—H12A	0.9300
C5—C6	1.350 (7)	C13—N3	1.369 (7)
C5—H5A	0.9300	C14—N3	1.462 (7)
C6—N1	1.336 (7)	C14—H14A	0.9600
C6—H6A	0.9300	C14—H14B	0.9600
C7—N2	1.318 (7)	C14—H14C	0.9600
C7—N3	1.357 (7)		
			
N1—C1—H1A	109.5	C10—C9—H9A	121.3
N1—C1—H1B	109.5	C8—C9—H9A	121.3
H1A—C1—H1B	109.5	C9—C10—C11	122.1 (6)
N1—C1—H1C	109.5	C9—C10—H10A	119.0
H1A—C1—H1C	109.5	C11—C10—H10A	119.0
H1B—C1—H1C	109.5	C12—C11—C10	121.1 (6)
N1—C2—C3	121.1 (5)	C12—C11—H11A	119.5
N1—C2—H2A	119.5	C10—C11—H11A	119.5
C3—C2—H2A	119.5	C11—C12—C13	116.7 (6)
C2—C3—C4	119.7 (6)	C11—C12—H12A	121.6
C2—C3—H3A	120.1	C13—C12—H12A	121.6
C4—C3—H3A	120.1	N3—C13—C12	131.6 (5)
C5—C4—C3	118.0 (5)	N3—C13—C8	105.5 (5)
C5—C4—C7	123.9 (5)	C12—C13—C8	122.9 (6)
C3—C4—C7	118.1 (5)	N3—C14—H14A	109.5
C6—C5—C4	119.6 (5)	N3—C14—H14B	109.5
C6—C5—H5A	120.2	H14A—C14—H14B	109.5
C4—C5—H5A	120.2	N3—C14—H14C	109.5
N1—C6—C5	121.8 (6)	H14A—C14—H14C	109.5
N1—C6—H6A	119.1	H14B—C14—H14C	109.5
C5—C6—H6A	119.1	C6—N1—C2	119.9 (5)
N2—C7—N3	114.0 (5)	C6—N1—C1	121.0 (5)
N2—C7—C4	121.4 (5)	C2—N1—C1	119.1 (5)
N3—C7—C4	124.6 (5)	C7—N2—C8	103.7 (5)
N2—C8—C13	110.2 (5)	C7—N3—C13	106.5 (5)
N2—C8—C9	130.0 (6)	C7—N3—C14	128.7 (5)
C13—C8—C9	119.8 (5)	C13—N3—C14	124.6 (5)
C10—C9—C8	117.4 (6)		
			
N1—C2—C3—C4	1.1 (9)	N2—C8—C13—C12	−176.0 (5)
C2—C3—C4—C5	−1.6 (9)	C9—C8—C13—C12	1.7 (8)
C2—C3—C4—C7	175.7 (5)	C5—C6—N1—C2	0.1 (9)
C3—C4—C5—C6	1.3 (9)	C5—C6—N1—C1	178.2 (6)
C7—C4—C5—C6	−175.7 (5)	C3—C2—N1—C6	−0.3 (9)
C4—C5—C6—N1	−0.6 (9)	C3—C2—N1—C1	−178.5 (6)
C5—C4—C7—N2	141.4 (6)	N3—C7—N2—C8	0.4 (6)
C3—C4—C7—N2	−35.7 (8)	C4—C7—N2—C8	−179.7 (5)
C5—C4—C7—N3	−38.7 (8)	C13—C8—N2—C7	−1.9 (6)
C3—C4—C7—N3	144.2 (5)	C9—C8—N2—C7	−179.3 (6)
N2—C8—C9—C10	176.4 (5)	N2—C7—N3—C13	1.2 (6)
C13—C8—C9—C10	−0.8 (8)	C4—C7—N3—C13	−178.7 (5)
C8—C9—C10—C11	−0.4 (8)	N2—C7—N3—C14	176.0 (5)
C9—C10—C11—C12	0.9 (9)	C4—C7—N3—C14	−3.8 (8)
C10—C11—C12—C13	−0.1 (8)	C12—C13—N3—C7	176.2 (5)
C11—C12—C13—N3	−179.4 (5)	C8—C13—N3—C7	−2.2 (5)
C11—C12—C13—C8	−1.2 (8)	C12—C13—N3—C14	1.1 (9)
N2—C8—C13—N3	2.6 (6)	C8—C13—N3—C14	−177.3 (5)
C9—C8—C13—N3	−179.7 (5)		
